# The Late Mr. William George Bunn

**Published:** 1911-09-30

**Authors:** 


### THE HOSPITAL WORLD.

### The late Mr. William George Bunn.

It is with sincere regret that we have to announce the death of Mr. W. G. Bunn, the late Secretary of the Hospital Saturday Fund. The sad event took place at his residence, 33 The Grove, Hammersmith, W., on Wednesday, September 20, 1911. Three weeks previously his illness took a serious turn; during the last fortnight he was unable to leave his bed, and was more or less delirious; on September 16 it was evident his strength was failing; and on September 20, at 7 p.m., he quietly passed away.

It may be recalled that, owing to failing health, Mr. Bunn resigned the secretaryship of the Fund on October 6, 1906. Since then his trouble, paralysis agitans, steadily sapped his strength, but he was able to do some useful work, in a quiet way, until recently. The funeral took place at Golder's Green, on Monday, September 25, at 12.30 p.m., when many representatives of the Fund and of the Friendly Societies, with which his life's work was associated, attended and paid honour to his memory.

Mr. W. G. Bunn, considering his social status, his education, and his opportunities, was a remarkable man. In recognition of his honorary services on behalf of the Fund he was elected in 1885 an invited member of the Board of Delegates of the Hospital Saturday Fund. He soon came to the front as an organiser, and in 1892 was elected its principal officer. Before this date, however, he had been officially connected with the Fund as its Organising Secretary. He joined the Fund when it was in storm and stress; he left it when it was enjoying peace and prosperity. Owing in a great measure to his persuasive eloquence and organising abilities the penny-a-week collection rapidly developed. During his tenure of office a great change was made in the system of collection; the annual street collection being discontinued in the year 1897. Other important developments advocated by Mr. Bunn were the provision of increased accommodation for convalescents at various homes, and for phthisical patients at hospitals and sanatoria; and of dental treatment and artificial teeth. In all the work of the Fund, Mr. Bunn strongly advocated self and mutual help.

Perhaps the three most telling traits of his character were his strong will, his cheerfulness, and his sense of brotherhood.

When he undertook a task, his indomitable will and energy never faltered. He talked, he wrote, he organised, he worked at it until his task was accomplished.

He was remarkable for his cheerfulness and geniality, and many of his most intimate friends believe the exercise of this quality was one of the causes of his success, and one of the lessons of his life. His clear judgment and good temper attracted the admiration of even his opponents, and he was never known willingly to hurt the susceptibilities of those who did not agree with him.

His sense of brotherhood in all humanity was certainly the most highly developed trait in his character. As Secretary of the Hospital Saturday Fund, and a prominent Friendly Society man, he was ever ready to help the sick and afflicted, to further the cause of the Friendly Societies.

We may give two instances of his work on behalf of others.

A dozen years ago some employers introduced the practice of making their employés join Shop Clubs as a condition of employment, and insisted on their leaving other Societies. Mr. Bunn's soul rebelled against this unwise and unjust procedure. He worked with splendid energy and courage until the Shop Clubs Act of 1902 was passed. Very few beyond his wife and family know of the circulars issued, the addresses given in London and the Provinces, the interviews with Members of Parliament, etc., before this end was achieved. Members of Friendly Societies have to thank Mr. Bunn more than any other man for their freedom to join any Friendly Society they think fit.

As Secretary of the Hospital Saturday Fund he was constantly realising the sad loss of life through want of adequate sanatorium accommodation. He set to work with his usual skill and energy, in order to convince workers for the Hospital Saturday Fund, members of Friendly Societies, and other bodies of the need of more sanatorium accommodation. In May, 1903, the matter was brought before the Board of Delegates of the Hospital Saturday Fund, and received earnest support; in October, 1905, the National Association for the Establishment and Maintenance of Sanatoria for Workers suffering from Tuberculosis was formally established; in November, 1905, he organised a most successful meeting of Friendly Society representatives at the Memorial Hall, Farringdon Street, E.C. The Hospital Saturday Fund encouraged the movement with a grant of £500. The work prospered, and the Benenden Sanatorium is the result. It is the opinion of all honorary workers of the Hospital Saturday Fund, who know the circumstances, that the establishment of the National Association, and the building of the Benenden Sanatorium, were due to the initiative of Mr. Bunn.

His long illness was a great strain on the bereaved wife and family. They ministered to his wants with the utmost solicitude and care. Such strenuous efforts as his for the sick and afflicted, for the community generally, cost the widow and family much—certainly more than others can realise. We tender to them our sincere and heartfelt sympathy in their heavy bereavement—a sympathy which will be shared in by all hospital workers in this country.

